# Efficacy of MEK inhibitors in Erdheim-Chester disease: impact of MAPK pathway pathogenic variants

**DOI:** 10.1038/s41375-025-02526-5

**Published:** 2025-02-11

**Authors:** Aldo A. Acosta-Medina, Jithma P. Abeykoon, Saurabh Zanwar, Gordon J. Ruan, Karen L. Rech, Aishwarya Ravindran, N. Nora Bennani, Caroline J. Davidge-Pitts, Matthew J. Koster, Jay H. Ryu, Mithun V. Shah, W. Oliver Tobin, Robert Vassallo, Jason R. Young, Ronald S. Go, Gaurav Goyal, on behalf of the Mayo Clinic - University of Alabama at Birmingham Histiocytosis Working Group, Aldo A. Acosta-Medina, Jithma P. Abeykoon, Saurabh Zanwar, Gordon J. Ruan, Karen L. Rech, Aishwarya Ravindran, N. Nora Bennani, Caroline J. Davidge-Pitts, Matthew J. Koster, Jay H. Ryu, Mithun V. Shah, W. Oliver Tobin, Robert Vassallo, Jason R. Young, Ronald S. Go, Gaurav Goyal

**Affiliations:** 1 Division of Hematology, Mayo Clinic, Rochester, MN USA; 2 Department of Laboratory Medicine and Pathology, Mayo Clinic, Rochester, MN USA; 3 Department of Pathology, University of Alabama at Birmingham, Birmingham, AL USA; 4 Division of Endocrinology, Diabetes and Nutrition, Mayo Clinic, Rochester, MN USA; 5 Division of Rheumatology, Mayo Clinic, Rochester, MN USA; 6 Division of Pulmonary and Critical Care Medicine, Mayo Clinic, Rochester, MN USA; 7 Mayo Clinic Center for Multiple Sclerosis and Autoimmune Neurology, Department of Neurology, Mayo Clinic, Rochester, MN USA; 8 Department of Radiology, Mayo Clinic, Jacksonville, FL USA; 9 Division of Hematology/Oncology, University of Alabama at Birmingham, Birmingham, AL USA; 10 Research Collaborator (limited tenure), Division of Hematology, Mayo Clinic, Rochester, MN USA

**Keywords:** Haematological cancer, Molecularly targeted therapy

Erdheim Chester disease (ECD) is a rare histiocytic neoplasm characterized by recurrent activating mutations in the mitogen-activated protein kinase – extracellular signal-regulated kinase (MAPK-ERK [*RAS-RAF-MEK-ERK]*) pathway, most commonly *BRAF*^V600E^ [1]. Vemurafenib was the first targeted therapy to be approved by the US-FDA for BRAF^V600E^-mutated ECD due to near-universal responses [2]. Subsequently, an MEK inhibitor (MEKi), cobimetinib, was evaluated and approved for histiocytic neoplasms regardless of the mutational status based on a small phase II trial showing an 89% overall response rate (ORR) [3]. Thereafter, BRAF- and MEK- inhibitors were incorporated into the various consensus guidelines as frontline therapy for this rare disease [4–6].

Despite rapid approval of cobimetinib, responses are not universal and there is a lack of data regarding predictors of response to MEKi in this rare disease. Our group recently reported a case of a patient with ECD whose disease was refractory to cobimetinib, and was found to harbor a *CSF1R* mutation [7]. Based on this finding as well as reports of other pathway mutations beyond MAPK-ERK in ECD [8], we hypothesized that the responses to MEK inhibitors in ECD may be dependent on the tumor mutational status. To test this hypothesis, we undertook the evaluation of outcomes of a large cohort of patients with ECD treated with MEKi outside of a clinical trial.

We queried the data from a prospective observational registry of patients with ECD from two tertiary care institutions. All cases of ECD were confirmed via correlation of clinical/radiographic and pathologic findings by the authors. Patients were included if treated with a MEKi (cobimetinib, trametinib, or binimetinib) between 2019 and 2021 and their MAPK-ERK pathway mutation status was known. This study was approved by the Institutional Review Board at both institutions.

Tumor MAPK-ERK pathway mutation status was determined by next-generation sequencing (NGS) using a 648 oncogene panel at diagnosis, mainly via the Tempus-xT® assay (Chicago, IL) with a threshold for variant report of 5%. If NGS data were unavailable, patients were only considered to have a known mutation status if *BRAF*^V600E^ immunohistochemistry (IHC) or *BRAF*^V600E/K^ allele-specific PCR was positive for a *BRAF*-specific mutation. Patients without available NGS due to low tumor cellularity and/or limited tissue availability and without *BRAF* mutations identified via IHC/PCR were considered to have incomplete genetic assessment and not included in our study. Patients were categorized as MAPK-ERK mutated if somatic alterations in the MAPK-ERK pathway were identified and all other patients were considered MAPK-ERK unmutated.

Primary endpoints included ORR and progression-free survival (PFS), defined as radiographic progression or death from any cause. Response assessment was conducted based on established F-18 fluorodeoxyglucose PET response criteria [5]. Survival curves were constructed using the Kaplan–Meier method and compared using the log-rank test. All time-to-event evaluations were calculated from the time of MEKi initiation. A secondary endpoint of interest was adverse events (AEs), graded using the National Cancer Institute Common Toxicity Criteria v5.0. All calculations were performed using SPSS^®^ version 28.0.1.1 (IBM Corp., Armonk, NY, USA).

A total of 20 patients with ECD were included in this study. The median age at diagnosis of ECD was 52 years (interquartile range [IQR] 35–67 years) and 10 (50%) were females. All but one patient in whom a *BRAF*^V600E^ was identified via IHC had a successful NGS. Among the 20 cases, 15 (75%) had pathogenic variants within the MAPK-ERK pathway, including *MAP2K1* (6/20, 30%), *BRAF*^V600E^ (2/20, 10%), *BRAF*-non-V600E alterations (6/20, 30%), and NRAS (1/20, 5%). The 5 cases included in the MAPK-ERK pathway unmutated group included those with alterations in *CSF1R* (2/5, 40%), *FLT3::MEF2C* (1/5, 20%), and two patients without variants identified via NGS. Patients with MAPK-ERK mutations were older at ECD diagnosis than the unmutated group (median 55 vs. 35 years; *p* = 0.006) but otherwise did not have significant differences at presentation. Detailed information regarding the mutation status for the cohort is presented in Supplementary Table 1.

The median follow-up time after MEKi initiation was 16 months (95% CI 10–31 months). Amongst the 20 patients, 26 separate instances of MEKi initiation were observed as five patients received two separate MEKi during their disease course and one patient underwent cobimetinib rechallenge after initial progression. MEKi was used as frontline therapy in 13 patients (65%) and as a median 3rd line therapy (range 2nd–6th line) in the rest of the cohort. The median time on MEKi was 8 months (IQR 5–20 months).

The most common initial MEKi used was cobimetinib (18/20, 90%) followed by trametinib (2/20, 10%). Of the six patients initiating a second MEKi, binimitenib was used in 3 (50%), cobimetinib in 2 (33%), and trametinib in 1 (17%). The most common initiating doses for MEKi included: cobimetinib 60 mg daily days 1–21 of each 28-day cycle (range 20 mg–60 mg), trametinib 2 mg daily (range 1 mg daily–2 mg daily), and binimetinib 45 mg twice daily (range 15 mg–45 mg).

The ORR with MEKi for the entire cohort was 80%, with 7 patients achieving a complete response (CR) and 9 patients achieving a partial response (PR). Extensive details regarding the patient follow-up course are presented in Fig. 1. Median PFS was not reached and the estimated 2-year PFS was 78% (95% CI 61%–99%). ORR was significantly higher amongst those with MAPK-ERK mutations compared with MAPK-ERK unmutated cases (93.3% vs. 40%, *p* = 0.032). Similarly, PFS was significantly prolonged in MAPK-ERK mutated compared with the unmutated subcohort (median PFS not reached vs. 7 months; *p* = 0.029) (Fig. 2). Notably, amongst the MAPK-ERK mutated cohort, the sole non-responder was a patient with a *BRAF*^V471F^ variant (patient #20). The remaining 3 patients not achieving a response belonged to the MAPK-ERK unmutated group, harboring *FLT3::MEF2C* fusion (*n* = 1) and *CSF1R* mutations (*n* = 2) all of whom had striking responses after initiation of FLT3-inhibitor (sorafenib) and CSF1R-inhibitor (pexidartinib), respectively).

**Fig. 1:**
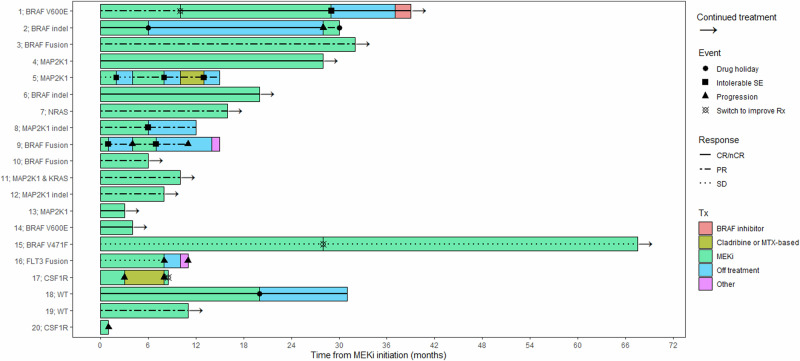
Swimmer’s plot detailing patient follow-up from the time of MEK inhibitor initiation.

**Fig. 2:**
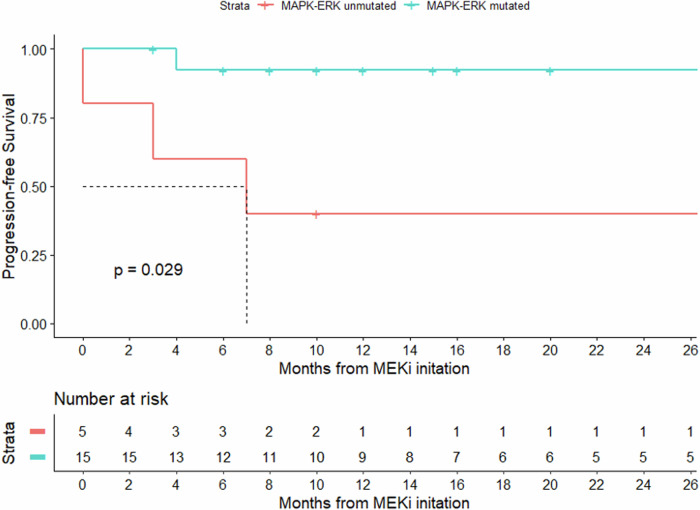
Progression-free survival for patients according to MAPK-ERK pathway mutation status.

All grade AEs were reported in 88% of MEKi treatment instances (23/26) with no differences noted between the specific MEKi agents (cobimetinib 89% vs. trametinib 66% vs. binimetinib 100%, *p* = 0.5). Most common AEs included diarrhea (*n* = 10), acneiform rash (*n* = 9), and fatigue (*n* = 7). Grade 3–4 AEs were experienced by 25% of patients (cobimetinib *n* = 3, trametinib *n* = 2) including diarrhea (*n* = 2) and one case each of thrombocytopenia, transaminitis, edema, and hypertension, respectively. Dose reduction of MEKi was undertaken in 11/26 instances (42%) of patients in an attempt to ameliorate symptoms (cobimetinib [45%] and binimetinib [67%]). Additionally, two patients switched to a second MEKi due to AEs.

All patients were alive at the last follow-up and 11/20 (55%) remained on MEKi. Reasons for discontinuation included intolerable AEs (*n* = 4, 20%), lack of response (*n* = 3, 15%), or drug holiday after response achievement (*n* = 2, 10%). Among the two patients undergoing a drug holiday, one had a persistent CR 11 months after discontinuation while the other experienced disease progression 22 months after MEKi discontinuation and had response recapture with resumption of MEKi.

This report is first-of-its-kind in demonstrating that among patients with ECD, the therapeutic response to MEKi is highly correlated with MAPK-ERK pathway mutation status. These findings are similar to those recently demonstrated in Rosai-Dorfman disease [9], and underscore the need to conduct tissue mutational assessment in each case to guide therapy. We observed a near-universal response among patients with pathogenic variants in the MAPK-ERK pathway. Additionally, pathogenic mutations outside of the MAPK-ERK pathway were identified in all 3 patients of the MAPK-ERK unmutated subgroup who did not respond to MEKi (two with *CSF1R* mutations and one with *FLT3::MEF2C* fusion) who subsequently responded to specific kinase inhibitors. Notably, one patient within the MAPK-ERK mutated group did not achieve a treatment response despite trials of trametinib and binimetinib (Patient #22). The mutation identified in this patient, *BRAF*^V471F^, is a class-II *BRAF* mutation which, due to intracellular signaling via RAF dimerization, is expected to exhibit intrinsic resistance to currently available BRAF and MEK inhibitors [10–13].

Strikingly, two patients without identified pathogenic variants through NGS experienced a therapeutic response to cobimetinib. While this may represent a failure of the NGS assay to pick up such mutations, alternative mechanisms of MEKi activity including inflammatory pathway regulation via effects on monocyte and dendritic cell differentiation may also play a relevant role leading to a response [9].

Although our study is limited by its small sample size, it represents the largest series of patients with ECD treated with MEKi outside of a clinical trial to date and our findings have critical implications for practice. The use of MEKi as a frontline strategy is justifiable in patients with ECD; however, clinicians must bear in mind that some cases may not be driven by MAPK-ERK pathway mutations and hence may not respond to MEK-inhibition and may need alternate treatments. Secondly, certain mutations even within the MAPK-ERK pathway, such as class-II *BRAF* mutations, may render the tumor resistant to BRAF- or MEK-inhibition. Thirdly, despite the FDA’s approval label specifying a daily dose of 60 mg for cobimetinib, our study shows a lower drug dose is often effective and better tolerated. This approach offers a more favorable toxicity profile, enhances patient tolerance, and extends therapy duration, thereby mitigating the necessity for drug discontinuation and reducing the risk of disease recurrence. Future research is needed to develop treatments, including novel agents that may be mutation agnostic and can be given for a finite duration leading to sustained remission.

## Supplementary information

Supplemental Material

## Data Availability

Limited dataset will be provided upon direct request to the authors. For original data request contact Go.Ronald@mayo.edu and ggoyal@uabmc.edu.
