# Supplementary material for: Efficacy of MEK inhibitors in Erdheim-Chester disease: impact of MAPK pathway pathogenic variants
Source: Leukemia. 2025 Feb 11;39(4):991–4. doi: 10.1038/s41375-025-02526-5 (PMC11976263; doi:10.1038/s41375-025-02526-5)
Supplement: Supplementary file 1 — Supplemental Material [file 41375_2025_2526_MOESM1_ESM.docx]

**Supplement**

Supplementary Table 1. Tumor tissue variant assessment tools and detected pathogenic variants

| **Patient ID** | | **Evaluation tools** | **Detected variants; Transcript ID** | **VAF [%]** | **Organ involvement** | | | | **Response to MEKi [1^st^; 2^nd^]** | |
| --- | --- | --- | --- | --- | --- | --- | --- | --- | --- | --- |
|  |  |  |  |  | **Skin** | **Bone** | **CNS** | **Cardiac** |  |  |
| *MAPK-ERK pathway mutated* | | | | | | | | | | |
| 1 | IHC | | *BRAF* ^V600E^ | - | - | - | Y | Y | PR; CR | |
| 2 | IHC & NGS | | *BRAF* ^N486_P490del^; NM_004333 | NR | - | - | Y | - | CR; PR | |
| 3 | IHC & NGS | | *UBTD2-BRAF* fusion | - | - | Y | Y | - | PR | |
| 4 | IHC & NGS | | *MAP2K1* ^K57N^; NM_002755 | 10.4 | - | - | - | Y | CR | |
| 5 | IHC & NGS | | *MAP2K1* ^Q56P^; NM_002755 | NR | - | - | - | - | SD; PR | |
| 6 | IHC & NGS | | *BRAF* ^T599_V600 DelInsRE^; NM_004333 | 3.3 | Y | - | Y | - | CR | |
| 7 | IHC & NGS | | *NRAS* ^Q61R^ ; NM_002524  *ASXL1* ^G646fs^; NM_015338 | 34.1  29.3 | - | Y | - | - | PR | |
| 8 | IHC & NGS | | *MAP2K1* ^E102_103del^; NM_002755 | 0.11 | Y | Y | - | Y | PR | |
| 9 | IHC & NGS | | *RNF11-BRAF* fusion | - | - | Y | Y | Y | PR; PR | |
| 10 | IHC & NGS | | *UBR2-BRAF* fusion | - | Y | Y | - | Y | PR | |
| 11 | IHC & NGS | | *MAP2K1* ^Y130C^; NM_002755  *KRAS* ^G12D^; NM_004985 | 4.3  6.3 | - | Y | - | - | PR | |
| 12 | IHC & NGS | | *MAP2K1* ^PE102_I103del^; NM_002755 | 7.1 | - | Y | - | - | PR | |
| 13 | IHC & NGS | | *MAP2K1* ^Q56P^; NM_002755 | 4.8 | - | Y | - | Y | CR | |
| 14 | IHC, as-PCR & NGS | | *BRAF* ^V600E^; NM_004333 | 5.2 | - | Y | Y | - | CR | |
| 15 | IHC, as-PCR & NGS | | *BRAF* ^V471F^ ; NM_004333  *NF1* ^c.1641+1G>A & c.7189+2T>G^; NM_000267  *MCL1* Amplification | NR | - | - | Y | - | SD; SD | |
| *MAPK-ERK pathway unmutated* | | | | | | | | | |  |
| 16 | IHC, as-PCR & NGS | | *FLT3-MEF2C* fusion | - | Y | Y | - | - | SD | |
| 17 | IHC & NGS | | *CSF1R* ^R549_E554delinsQ^; NM_005211 | 27.4 | - | Y | Y | - | PD; NA | |
| 18 | IHC & NGS | | No pathogenic variant identified | - | Y | Y | - | - | CR | |
| 19 | IHC & NGS | | No pathogenic variant identified | - | - | Y | - | Y | PR | |
| 20 | IHC & NGS | | *CSF1R* ^S560_P566del^; NM_005211  *CSF1R* ^S938_Y969del^; NM_005211 | 31.9  14.3 | - | - | Y | - | PD | |
| Abbreviations – as-PCR: allele-specific polymerase chain reaction, CR: complete response; IHC: immunohistochemistry, MEKi: MEK inhibitor, NA: not assessed; NGS: next-generation sequencing; NR: not reported; PD: progressive disease; PR: partial response; SD: stable disease VAF: variant allele frequency | | | | | | | | | | |
